# The affordable medicines facility - malaria in Ghana: baseline and endline survey findings

**DOI:** 10.1186/1475-2875-11-S1-P109

**Published:** 2012-10-15

**Authors:** John H Amuasi, Samuel Blay Nguah, Daniel Ansong, Graciela Diap

**Affiliations:** 1 University of Minnesota School of Public Health, Division of Health Policy and Management, Minneapolis, MN 55455, USA; 2 Komfo Anokye Teaching Hospital, Kumasi, Ghana; 3 Drugs for Neglected Diseases initiative, Geneva, Switzerland; 4 The AMFm Independent Evaluation team: ICF International (Fred Arnold, Yazoume Ye, Ruilin Ren) and London School of Hygiene and Tropical Medicine (Kara Hanson, Catherine Goodman, Sarah, Tougher, Barbara Willey, Andrea Mann

## 

The Affordable Medicines Facility - malaria (AMFm), hosted by the Global Fund to Fight AIDS, Tuberculosis and Malaria, is a financing mechanism which subsidizes quality-assured Artemisinin-based Combination Therapies (ACTs) for distribution to the public and private sectors, complemented by supporting interventions to promote rational drug use. AMFm has been in operation since mid-2010 in eight national-scale operational pilots in Ghana, Kenya, Madagascar, Niger, Nigeria, Tanzania mainland, Uganda and Zanzibar. By March 2012, over 220 million co-paid ACT treatment doses had been ordered.

The Independent Evaluation (IE) of AMFm Phase 1 was commissioned by the Global Fund to assess the impact of AMFm on availability, price, market share and use of quality-assured ACTs in all the operational pilots as part of evidence gathering needed to inform decisions regarding the future of the AMFm. The assessment is based on a pre- and post-test design with detailed documentation of the implementation process and context, treating each pilot independently. In each pilot, a nationally representative survey of outlets stocking antimalarial medicines was conducted at the baseline (2009/10) and the endline (2011).

In Ghana, the IE was carried out by a team comprising collaborators from the Komfo Anokye Teaching Hospital, Drugs for Neglected Diseases *initiative*, ICF International and London School of Hygiene and Tropical Medicine. As part of the IE, national level baseline and endline outlet surveys were conducted in Ghana involving the collection and analysis of primary data to answer three questions related to the availability, affordability and market share of quality-assured ACTs using a cluster sampling approach. In-depth key informant interviews were also conducted to provide the necessary context information to help in the interpretation of the survey results.

In Ghana, 1,241 and 1,093 outlets were enumerated for the baseline and endline outlet surveys respectively. For the baseline survey, 57.3% (CI: 50.5-64) of outlets with any antimalarials in stock at the time of the survey visit carried artemisinin monotherapies, while only 30.7% (CI: 26.1-35.8) of interviewed outlets had quality-assured ACTs in stock at the time of the survey visit. At baseline, the median cost to patients of one adult equivalent treatment dose of quality-assured ACTs was US$ 3.42 (IQR: 2.4-7.53) for 1,092 products. Detailed results on changes in quality-assured ACT availability, price and market share over a 14-month period between baseline and endline surveys in Ghana will be presented.

